# Increased temperature and CO_2_ alleviate photoinhibition in *Desmarestia anceps:* from transcriptomics to carbon utilization

**DOI:** 10.1093/jxb/erx164

**Published:** 2017-06-01

**Authors:** Concepción Iñiguez, Sandra Heinrich, Lars Harms, Francisco J L Gordillo

**Affiliations:** 1 University of Malaga, Department of Ecology, Faculty of Sciences, Boulevard Louis Pasteur s/n, Málaga, Spain; 2 University of Hamburg, Ohnhorst Str., Hamburg, Germany; 3 Alfred-Wegener-Institute, Helmholtz Centre for Marine and Polar Research, Am Handelshafen, Bremerhaven, Germany

**Keywords:** Antarctica, carbon-concentrating mechanisms, carbon dioxide, global change, macroalgae, ocean acidification, photosynthesis, seaweeds, transcriptome, warming

## Abstract

Ocean acidification and warming are affecting polar regions with particular intensity. Rocky shores of the Antarctic Peninsula are dominated by canopy-forming Desmarestiales. This study investigates the physiological and transcriptomic responses of the endemic macroalga *Desmarestia anceps* to a combination of different levels of temperature (2 and 7 °C), dissolved CO_2_ (380 and 1000 ppm), and irradiance (65 and 145 µmol photons m^−2^ s^−1^). Growth and photosynthesis increased at high CO_2_ conditions, and strongly decreased at 2 °C plus high irradiance, in comparison to the other treatments. Photoinhibition at 2 °C plus high irradiance was evidenced by the photochemical performance and intensive release of dissolved organic carbon. The highest number of differentially regulated transcripts was observed in thalli exposed to 2 °C plus high irradiance. Algal ^13^C isotopic discrimination values suggested an absence of down-regulation of carbon-concentrating mechanisms at high CO_2_. CO_2_ enrichment induced few transcriptomic changes. There was high and constitutive gene expression of many photochemical and inorganic carbon utilization components, which might be related to the strong adaptation of *D. anceps* to the Antarctic environment. These results suggest that increased temperature and CO_2_ will allow *D. anceps* to maintain its productivity while tolerating higher irradiances than at present conditions.

## Teaser

The expected scenario of increased temperature and CO_2_ concentration in the Antarctic Peninsula area will allow the forest-forming seaweed *Desmarestia anceps* to tolerate higher irradiances while maintaining its productivity.

## Introduction

Global change is affecting polar regions to a larger extent than any other region on Earth (Intergovernmental Panel on Climate Change, 2013). In the Southern Hemisphere, the strongest rates of atmospheric warming are occurring in the western and northern parts of the Antarctic Peninsula and its surrounding islands (Larsen *et al.*, 2014). In coastal areas on King George Island, summer temperatures of the upper 30 m of seawater have risen by 0.32 °C per decade since 1991 (Schloss *et al.*, 2012), and it is predicted that the temperature of the Southern Ocean will continue rising (Convey *et al.*, 2009).

In addition to warming, polar waters are particularly vulnerable to ocean acidification (OA) due to the increased solubility of CO_2_ in cold waters—an effect that is further amplified by decreased salinity resulting from ice melting (Midorikawa *et al.*, 2012).

The retreat of sea-ice cover in coastal areas as a consequence of global warming will result in increased irradiance levels in the water column. However, a recent reduction in phytoplankton productivity in the northern Antarctic Peninsula region has been observed as a consequence of reduced ice cover (Montes-Hugo *et al.*, 2009), probably owing to less stratified conditions in response to an increase in wind mixing. This decline in phytoplankton may result in a significant rise in subtidal irradiance in areas of low turbidity.

Large perennial macroalgal species of the order Desmarestiales dominate the sublittoral hardbottom zones of the Antarctic Peninsula coastline and the surrounding islands (Klöser *et al.*, 1994; Brouwer *et al.*, 1995), structuring highly productive underwater forests, which replace the ecological function of kelps in temperate and Arctic waters (Clayton, 1994). The most common species are *Desmarestia anceps*, *Desmarestia menziesii*, and *Himantothallus grandifolius*, all of which are endemic to Antarctica (Wiencke and Clayton, 2002). *Desmarestia anceps* generally grows in the mid-sublittoral zone, down to 15–20 m depth, and typically occurs in moderately exposed sites (Quartino *et al.*, 2001). The sporophytes of this species exhibit maximum growth rates at 0–5 °C (Wiencke and tom Dieck, 1989) and are strongly shade-adapted, showing very low light requirements for photosynthesis and growth (Wiencke, 1990).

Increased CO_2_ concentrations are expected to have a fertilization effect on marine autotrophs by increasing gross primary production (Hein and Sand-Jensen, 1997), since ribulose-1,5-bisphosphate carboxylase/oxygenase (Rubisco) is supposed to be undersaturated at the present CO_2_ concentrations in most photosynthetic marine organisms (Raven and Beardall, 2003). However, the photosynthetic response to increased CO_2_ also depends on the presence of carbon-concentrating mechanisms (CCMs) that enhance and often saturate the photosynthetic carbon demand by increasing the CO_2_ concentration around Rubisco. CCMs consist of an active inﬂux of CO_2_ and/or HCO_3_^−^ at the plasma membrane and/or plastid envelope membrane (Maberly *et al.*, 1992; Raven *et al.*, 2002*b*; Raven and Beardall, 2003). CCMs are energetically expensive, so at a sufficient CO_2_ concentration, a down-regulation of their activity can occur, leading to energy saving (Johnston and Raven, 1991; Magnusson *et al.*, 1996; Yang and Gao, 2012), which may result in an enhancement of growth in some cases (Gordillo *et al.*, 2001; Iñiguez *et al.*, 2015). Nevertheless, other macroalgal species have not shown a down-regulation of CCMs at increased CO_2_ levels (Fernández *et al.*, 2015; Rautenberger *et al.*, 2015).

It has been proposed that polar macroalgae might have a lower requirement for CCM operation due to the higher solubility of CO_2_ and a suspected increase in Rubisco affinity for CO_2_ and in the selectivity factor of Rubisco for CO_2_ relative to O_2_ (*S*_C/O_) in cold waters (Raven *et al.*, 2002*a*). However, all Arctic seaweeds analysed to date have shown high external carbonic anhydrase (CA) activities (Gordillo *et al.*, 2006), and some studies based on algal ^13^C isotopic discrimination values (δ^13^C_alga_), which are used as a proxy for bicarbonate uptake (Maberly *et al.*, 1992), have revealed that most polar macroalgae possess the ability to use HCO_3_^−^ for photosynthesis (Wiencke and Fischer, 1990; Fischer and Wiencke, 1992). Additionally, Beardall and Roberts (1999) reported kinetics of dissolved inorganic carbon (DIC)-dependent oxygen evolution for Antarctic seaweeds that were consistent with the presence of an active CCM. Previously published δ^13^C_alga_ values for *D. anceps* are inconclusive with respect to CCM operation, ranging from –25.3‰ (Dunton, 2001) to –30.68‰ (Fischer and Wiencke, 1992). This variability can be explained by the fact that expression of CCMs is highly regulated by a number of environmental factors, that is, light, dissolved CO_2_ concentration, and temperature (Giordano *et al.*, 2005). Seasonal and spatial variability in the δ^13^C_alga_ value of kelp forest across depth gradients has been reported by Hepburn *et al.* (2011). Thus, the more negative δ^13^C_alga_ values found in *D. anceps* may reflect its strong shade adaptation in sporophytes of deeper areas and/or during the autumn/winter months, reflecting low or no CCM activity (Kübler and Raven, 1994), while a higher demand for CCMs at higher irradiances might be responsible for the less negative δ^13^C_alga_ values.

In addition to photosynthesis and CCM operation, other physiological processes might be directly or indirectly affected by OA. Respiration and dissolved organic carbon (DOC) release, which represent the main carbon losses in algae, have been shown to be altered by increased CO_2_ conditions in some seaweed species (Gordillo *et al.*, 2001; Iñiguez *et al.*, 2015), determining the carbon balance of the whole plant under a given environmental condition. These processes, which have usually been overlooked in studies of this type, have been shown to be of prime relevance in defining the effects of environmental factors on growth performance.

Previous studies have revealed that the physiological response to OA and to warming separately might be modified by the interaction of the two stressors, producing antagonistic or synergistic effects. Holding *et al.* (2015) showed that Arctic phytoplanktonic primary production was enhanced at high CO_2_ levels, but only when exposed to low temperatures and not under warming conditions. Conversely, the growth and photosynthesis of the kelp *Macrocystis pirifera* were not affected by CO_2_ and were significantly reduced by elevated temperature, but a positive response was observed when the alga was grown under elevated temperature in combination with elevated CO_2_ relative to ambient conditions (Brown *et al.*, 2014).

Likewise, the interaction with light has been shown to be key to the response of macrophytes to global change, as inorganic carbon acquisition and assimilation are strongly dependent on light energy availability (Hepburn *et al.*, 2011; Gao *et al.*, 2012). Lower irradiances may reduce the potential effects of OA and warming. This has been reported for the red macroalga *Gracilaria lemaneiformis* (Zou and Gao, 2009), and for the coccolithophore *Emiliania huxleyi* (Feng *et al.*, 2008), with both species showing a significant increase in growth rate at elevated CO_2_ but only at intermediate to saturating irradiance and not at low irradiance. Conversely, CO_2_ can also affect the threshold at which irradiance becomes excessive, as was shown for the chlorophyte *Dunaliella tertiolecta*, which exhibited a higher physiological tolerance for excessive irradiance conditions at elevated CO_2_ compared with current CO_2_ levels (García-Gómez *et al.*, 2014).

Little is known about the molecular mechanisms involved in these physiological acclimation responses in algae. Extensive gene expression analyses, after acclimation to increased CO_2_, were conducted for *E. huxleyi* (Rokitta *et al.*, 2012; Benner *et al.*, 2013), and for the diatoms *Phaeodactylum tricornutum* (Li *et al.*, 2015) and *Thalassiosira pseudonana* (Crawfurd *et al.*, 2011; Hennon *et al.*, 2015), but this type of information has not been published for seaweeds.

The aim of this study was to analyse the physiological response of the ecologically relevant Antarctic endemic macroalga *D. anceps* to likely future conditions of increased CO_2_ and temperature at saturating and photoinhibitory irradiance, and to investigate the molecular mechanisms underlying physiological acclimation to near-future scenarios. Growth, photosynthesis, respiration, DOC release, CCM operation (including CA activity), and elemental composition were studied, in addition to an analysis of a RNA-Seq dataset. These results provide novel and valuable data on the biochemical regulation and physiological functioning of *D. anceps* in response to the main environmental factors related to global change.

## Materials and methods

### Plant material

Young sporophytes of *Desmarestia anceps* Montagne were raised from Alfred Wegener Institute (AWI) stock cultures of female (culture number: 3084) and male (culture number: 3064) gametophytes, established from spores of fertile sporophytes collected at Potter Cove (King George Island, South Shetland Islands, Antarctica; 62°14ʹS, 58°38ʹW) using the cultivation methods described by Wiencke and tom Dieck (1989). Thalli were developed in a culture room at 0 ± 1 °C, using sterile 0.2 µm-filtered seawater (FSW) enriched with unbuffered nutrients, after Provasoli (1968). The day length was adjusted weekly, mimicking the seasonal variation at King George Island (Wiencke, 1990). Sporophytes were transferred to 5 l beakers at a photon ﬂuence rate (PFR) of 50–55 µmol photons m^−2^ s^−1^ provided by white light fluorescent tubes (L58W/965; Osram, Germany); PFR was measured in the water in the middle of the beaker using a spherical micro quantum sensor (US-SQS/L; Walz, Germany) connected to a radiometer (LiCor-250A; Li-Cor Biosciences, USA).

### Experimental setup

Thalli 15–20 cm in length were incubated for 13 days at two different CO_2_ concentrations, 380 ppm (A) and 1000 ppm (C), combined with two different temperatures, 2 °C and 7 °C, and two different irradiance levels, 65 (LL) and 145 µmol photons m^−2^ s^−1^ (HL). The chosen irradiances represented optimum and photoinhibitory irradiances, respectively, for the growth of juvenile sporophytes of *D. anceps* at 0 °C (Wiencke and Fischer, 1990). Experiments were carried out in temperature-controlled rooms (2 ± 1 °C and 7 ± 1 °C) with a 18:6 h light:dark photoperiod, using glass beakers containing 1.8 l FSW. Six replicate beakers, each containing ~1 g fresh weight (FW) thallus tissue, were used for each treatment. Beakers were aerated continuously with artificial air (20% oxygen, 80% nitrogen) with either 380 or 1000 ppm CO_2_, generated by a gas-mixing device (HTK GmbH, Hamburg, Germany), at 600 ml min^−1^. The two CO_2_ conditions were verified by measuring seawater pH (NBS scale) and determining total alkalinity by potentiometric titrations (Gran, 1952) every other day. CO_2_ speciation was calculated using the CO2calc Package (Robbins *et al.*, 2010), with the CO_2_ acidity constants of Mehrbach *et al.* (1973) and the CO_2_ solubility coefficient of Weiss (1974) (see Supplementary Table S1 at *JXB* online). Three days of pre-acclimation were applied before the experiments to avoid the interference of transient responses. FSW aerated with the different gas mixtures for 24 h before use was exchanged every 4 days. The physiological measurements were conducted at the end of the incubation period, using sterile FSW pre-equilibrated at either 390 or 1000 ppm CO_2_ at 2 or 7 °C. Sporophytes were frozen in liquid nitrogen and stored at –80 °C for further analyses. Growth rate was calculated from the initial and final FW, assuming exponential growth.

### Chlorophyll fluorescence

Measurement of the optimal quantum yield for photosystem II (PSII) fluorescence (*F*_v_/*F*_m_) after 15 min of incubation in darkness, followed by rapid light curves (RLC) consisting of eight increasing white light intensities (20 s of exposure to each intensity), was done using a PAM 2100 (Walz, Effeltrich, Germany). The electron transport rate between PSII and photosystem I (ETR) at each irradiance was calculated as described by Iñiguez *et al.* (2015). The thallus absorptance was also analysed in order to calculate absolute instead of relative ETR values. The following photosynthetic parameters were obtained from the fitting of the RLC to the non-linear least squares regression model by Eilers and Peeters (1988): maximum electron transport rate (*ETR*_max_), photosynthetic light-harvesting efficiency (α), saturating irradiance (*E*_k_), and irradiance at which chronic photoinhibition begins (*E*_0pt_).

### Photosynthesis, respiration, and use of CA inhibitors

Net photosynthesis (NPS) at culture PFR provided by white light LED lamps, as well as dark respiration, were estimated by oxygen evolution using a Clark-type oxygen electrode (5331; Yellow Springs Instruments, USA), as described by Iñiguez *et al.* (2015).

The effect of the CA inhibitors 6-ethoxyzolamide (EZ; Sigma-Aldrich, Spain) and dextran-bound sulfonamide (DBS; Ramidus AB, Sweden) on NPS was also tested under culture PFR. Stock solutions of these inhibitors were prepared in 0.05 N NaOH and were added to the chambers to a final concentration of 200 µM (Flores-Moya and Fernández, 1998). The same sample (100–150 mg FW) was used for all oxygen evolution measurements, by consecutively determining dark respiration, NPS, NPS after inhibition by DBS, and NPS after inhibition by EZ, changing the FSW medium between each measurement to prevent oversaturation of oxygen. Rates were calculated approximately 10 min after the addition of the inhibitors, when the linear slope of [O_2_]/s was stable.

### Total carbon and nitrogen content

Total internal C and N content was determined from freeze-dried tissue samples after homogenization with a Mixer Mill (MM 400; Retsch), using a C:H:N elemental auto-analyser (Perkin-Elmer 2400CHN) by the difference-on-ignition method (Kristensen and Andersen, 1987).

### Stable isotopic determination

The ^13^C isotopic discrimination in the algal samples (δ^13^C_alga_) was determined by mass spectrometry using a DELTA V Advantage (Thermo Electron Corporation, USA) Isotope Ratio Mass Spectrometer (IRMS) connected to a Flash EA 1112 CNH analyser, as described by Iñiguez *et al.* (2016). The ^13^C isotopic discrimination of the dissolved inorganic carbon found in the medium (δ^13^C_DIC_) was measured with the same IRMS connected to a GasBench II (Thermo Electron Corporation) system, using 20 ml FSW collected from each cylinder, previously filtered (Whatman GF/F). The δ^13^C_alga_ was corrected with the δ^13^C_DIC_ values from the medium, since the CO_2_ source used in the experiment for the CO_2_-enriched treatment came from previously fixed CO_2_ that had been already discriminated.

### Dissolved organic carbon

Samples for the determination of DOC in the medium were taken at the beginning and the end of the incubation period, and before and after each water change. After filtration of 20 ml FSW (Whatman GF/F), the water samples were acidified by adding 100 µl 0.5 N HCl and kept in glass vials at 4 °C until analysis by an automated system (TOC-L CSN; Shimadzu Corporation, Japan), according to the manufacturer’s protocols. All the materials used for sampling, filtration, and storage of the samples were previously cleaned with 5% HCl. Filters and glass vials were pre-combusted at 500 °C for 5 hours to eliminate any organic contamination.

### Pigment content

Pigments (Chl *a*, Chl *c*, and total carotenoids) were extracted in *N*,*N*-dimethylformamide. After an incubation period of 24 h at 4 °C in darkness, the concentrations were determined spectrophotometrically. For Chl *a* and Chl *c* contents, the methodology of Henley and Dunton (1995) was followed. Crude estimations of total carotenoids were calculated using the equation proposed by Parsons *et al.* (1984).

### Physiological data analyses

Significance of differences (*P*<0.05, *n*=6) between the different treatments was tested using a three-factorial analysis of variance (ANOVA), after normality (Shapiro-Wilk test) and homogeneity of variances (Cochran’s test) were confirmed. Post-hoc comparisons were performed by Fisher’s least significant difference (LSD) test (*P*<0.05). All statistical analyses were performed using Statistica software v.7 (StatSoft Inc., USA).

### RNA extraction, Illumina sequencing, and data processing

Total RNA extraction was conducted by the method of Heinrich *et al.* (2012). RNA quality was analysed by microfluidic electrophoresis with the Agilent 2100 Bioanalyzer (Agilent Technologies, Germany). cDNA library construction and sequencing was performed by using a Eurofin MWG (Ebersberg, Germany). In brief, mRNA was isolated using oligo-dT beads followed by fragmentation, random-primed cDNA synthesis, and Illumina-compatible adaptor ligation. Sequencing was carried out on an Illumina Hiseq 2500 instrument with three biological replicates per treatment. Raw reads were quality controlled by FastQC v. 0.10.01 (Babraham Institute, Cambridge, UK) and quality filtered using Trimmomatic v. 0.32 (Bolger *et al.*, 2014). Quality filtering was performed using the following parameters: leading 3, trailing 3, sliding window 4:15, minlen 30. The cleaned raw data were deposited in the European Nucleotide Archive (ENA) at the European Molecular Biological Laboratory–European Bioinformatics Institute under study accession number PRJEB18576 (http://www.ebi.ac.uk/ena/data/view/PRJEB18576).

Short reads of each sample were separately aligned against the *de novo* reference transcriptome (raw data: ENA PRJEB18576), using Bowtie v. 1.0.0 (Langmead *et al.*, 2009). Relative abundances were estimated by RSEM v. 1.2.11 (Li and Dewey, 2011) and genes were analysed for differential expression using edgeR (Robinson *et al.*, 2010), with a standard level of *P*≤0.01 and a fold change of at least 2 indicating significance. Tools were executed using the Trinity package release 2014-07-17 (Grabherr *et al.*, 2011). To detect gene expression changes associated with acclimation responses, pairwise comparisons of each treatment with the control treatment (2 °C-LLA) were performed. For exploring constitutively expressed transcripts within the control, normalized read counts, given as transcripts per kilobase million, were analysed. Functional annotation was performed using the Trinotate functional annotation suite 2014-07-08 (Grabherr *et al.*, 2011). To investigate the function of significantly up- and down-regulated genes in comparison to the control, Gene Ontology (GO) enrichments were conducted using GOseq (Young *et al.*, 2010). Enriched GO terms were summarized with the CateGOrizer (Zhi-Liang *et al.*, 2008) using the EGAD2GO classification file.

## Results

### Physiological performance

The physiological performance and elemental composition of *D. anceps* were highly sensitive to changes in irradiance, temperature and, to a lesser extent, CO_2._ Furthermore, a significant interaction between irradiance and temperature was observed in almost all variables analysed (Supplementary Table S2).

**Fig. 1.**
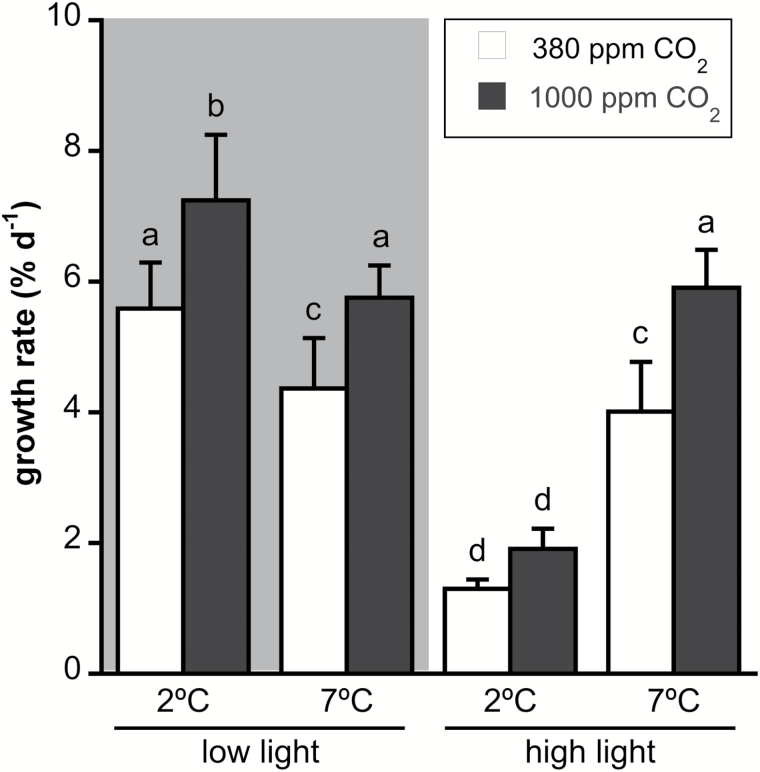
Growth rate (expressed as % d^−1^) of *Desmarestia anceps* during 12 days of culture at different CO_2_ levels (380 or 1000 ppm), temperatures (2 or 7 °C), and irradiance conditions (65 μmol photons m^−2^ s^−1^ or 145 μmol photons m^−2^ s^−1^). Values are mean±SD (*n*=6). Significant differences (*P*<0.05) revealed by Fisher’s LSD test following a three-way ANOVA (CO_2_, temperature, and light) are indicated by different letters.

Growth rate was significantly affected by the three factors and by the interaction between irradiance and temperature (Supplementary Table S2). High CO_2_ produced a significant increase of ~30–40% in growth rate in all conditions except for 2 °C-HL (Fig. 1). HL produced a strong decrease of more than two-thirds in the growth rate at 2 °C relative to LL, while there were no significant changes in the growth rates at 7 °C between the two irradiance conditions. Moreover, the growth rate of 7 °C-LL thalli was significantly lower than that of 2 °C-LL thalli, decreasing from 5.6 to 4.4% d^−1^ at present CO_2_ levels and from 7.2 to 5.6% d^−1^ at high CO_2_ (Fig. 1).

**Fig. 2.**
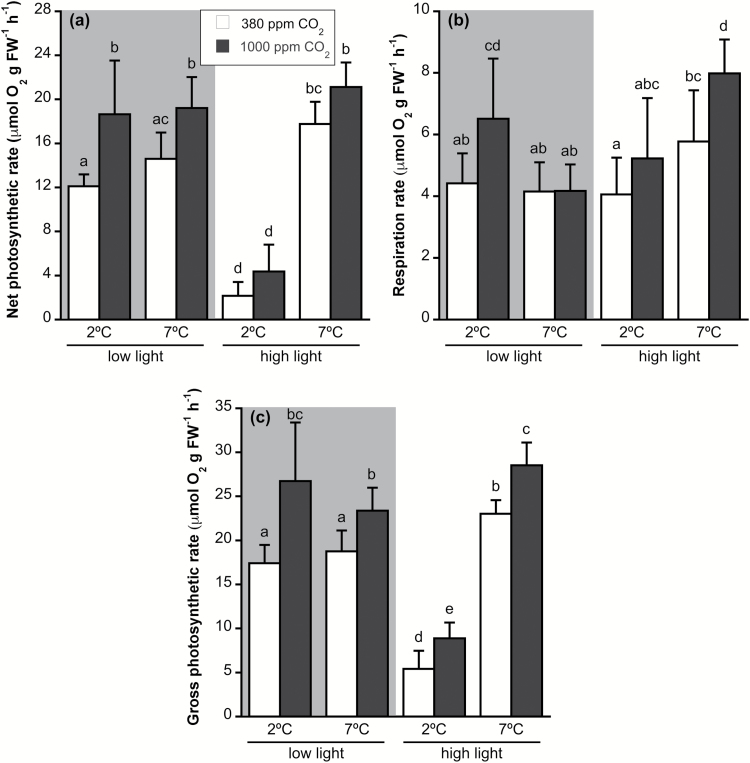
(a) Net photosynthetic rate, (b) dark respiration rate, and (c) gross photosynthetic rate measured by oxygen evolution of *Desmarestia anceps* after 12 days of culture at different CO_2_ levels (380 or 1000 ppm), temperatures (2 or 7 °C) and irradiance conditions (65 μmol photons m^−2^ s^−1^ or 145 μmol photons m^−2^ s^−1^). Values are mean±SD (*n*=6). Significant differences (*P*<0.05) revealed by Fisher’s LSD test following a three-way ANOVA (CO_2_, temperature, and light) are indicated by different letters.

Photosynthesis followed the same pattern as growth rate. High CO_2_ provoked a general significant enhancement of gross photosynthesis, whereas the increase of net photosynthesis at high CO_2_ levels was significant only under LL conditions (Fig. 2a, c). HL caused a decrease of 65–75% in net and gross photosynthesis at 2 °C, while at 7 °C, HL produced a significant enhancement of 20–30% in gross photosynthesis. Higher temperature did not alter net or gross photosynthesis at LL.

Respiration rate was significantly affected by increased CO_2_ and irradiance, and by the interaction between irradiance and temperature, but not by temperature alone (Supplementary Table S2). High CO_2_ caused a significant increase in respiration rate by 25–30% at 2 °C-LL and 7 °C-HL, although no significant change was observed for 7 °C-LL and 2 °C-HL (Fig. 2b). HL led to a significant increase in respiration rates at 7 °C at high CO_2_ conditions, while increased temperature enhanced respiration rates at HL.

**Fig. 3.**
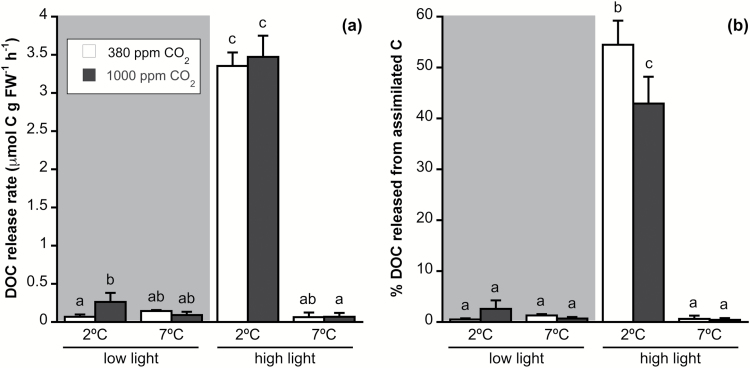
(a) Dissolved organic carbon (DOC) release rate and (b) percentage of DOC released from assimilated C of *Desmarestia anceps* after 12 days of culture at different CO_2_ levels (380 or 1000 ppm), temperatures (2 or 7 °C), and irradiance conditions (65 μmol photons m^−2^ s^−1^ or 145 μmol photons m^−2^ s^−1^). Values are mean±SD (*n*=6). Significant differences (*P*<0.05) revealed by Fisher’s LSD test following a three-way ANOVA (CO_2_, temperature, and light) are indicated by different letters.

DOC release rate was affected by increased irradiance and temperature and by the interaction between the two factors, but not by CO_2_ (Supplementary Table S2). However, DOC release calculated as a percentage of assimilated C was significantly affected by all factors and their interactions. Both ways of calculating DOC release showed a significant increase of >90% at 2 °C-HL compared with the rest of the treatments (Fig. 3). High CO_2_ produced a significant decrease in the percentage of assimilated C being released as DOC at 2 °C-HL, from 54 to 43%.

**Fig. 4.**
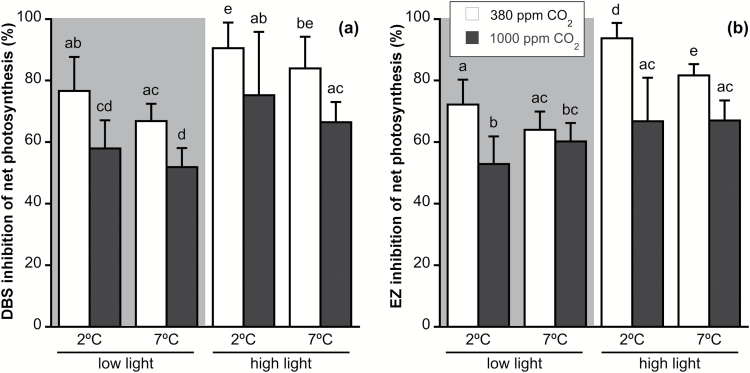
(a) Dextran-bound sulfonamide (DBS) and (b) ethoxyzolamide (EZ) inhibition of net photosynthetic rate of *Desmarestia anceps* after 12 days of culture at different CO_2_ levels (380 or 1000 ppm), temperatures (2 or 7 °C), and irradiance conditions (65 μmol photons m^−2^ s^−1^ or 145 μmol photons m^−2^ s^−1^). Values are mean±SD (*n*=6). Significant differences (*P*<0.05) revealed by Fisher’s LSD test following a three-way ANOVA (CO_2_, temperature, and light) are indicated by different letters.

Photosynthesis was inhibited by the CA inhibitors DBS, which inhibits only external CAs, and EZ, which inhibits both external and internal CAs (Moroney *et al.*, 1985; Fig. 4). Both treatments led to similar results, causing a reduction of 50–90% of net O_2_ production. DBS inhibition was significantly affected by CO_2_, irradiance, and temperature, but not by any of their interactions. EZ inhibition was influenced by CO_2_ and irradiance, and by the interaction of CO_2_ and temperature, but not by temperature alone (Supplementary Table S2). Elevated CO_2_ significantly decreased DBS inhibition of net photosynthesis by 15–20% in all cases, while HL conditions produced a general increase in DBS photosynthetic inhibition.

**Fig. 5.**
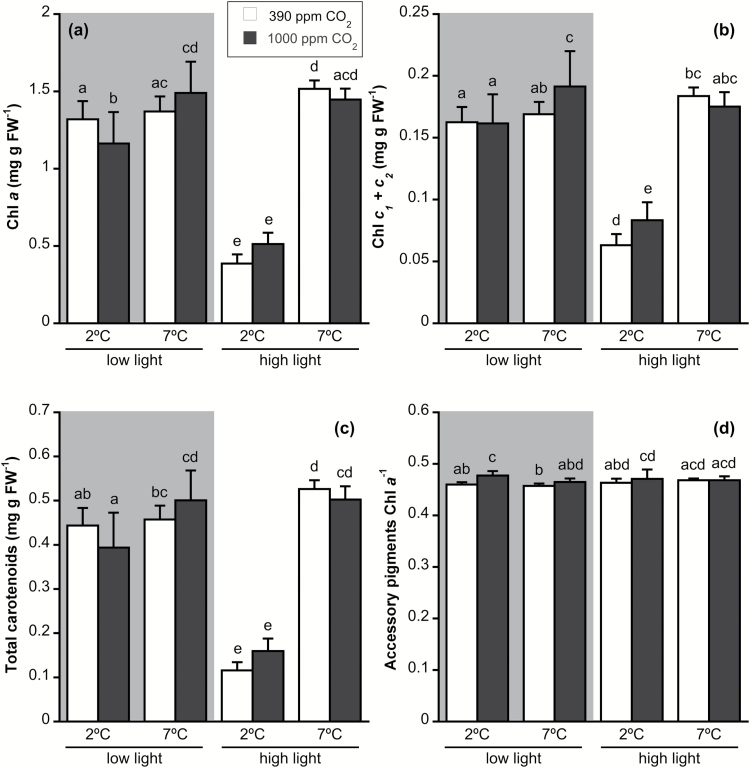
(a) Chl *a* content, (b) Chl *c* content, (c) relative total carotenoid content, and (d) accessory pigments Chl *a*^−1^ of *Desmarestia anceps* after 12 days of culture at different CO_2_ levels (380 or 1000 ppm), temperatures (2 or 7 °C), and irradiance conditions (65 μmol photons m^−2^ s^−1^ or 145 μmol photons m^−2^ s^−1^). Values are mean±SD (*n*=6). Significant differences (*P*<0.05) revealed by Fisher’s LSD test following a three-way ANOVA (CO_2_, temperature, and light) are indicated by different letters.

Chl *a*, Chl *c*, and total carotenoid contents were affected by irradiance and temperature, and by the interactions of irradiance and temperature and of all factors, but not by CO_2_ alone (Supplementary Table S2). Chl *a*, Chl *c*, and total carotenoid contents were significantly reduced at 2 °C-HL (Fig. 5). Higher CO_2_ caused a decrease of Chl *a* content at 2 °C-LL and an increase of Chl *c* at 7 °C-LL and 2 °C-HL. Furthermore, increased temperature led to a higher pigment content at LL at elevated CO_2_. The ratio accessory pigments Chl *a*^−1^ was significantly influenced by CO_2_, with elevated CO_2_ producing a significant increase at 2 °C-LL.

All parameters obtained from Chl *a* fluorescence measurements were significantly affected by irradiance and by the interaction of irradiance and temperature (Supplementary Table S2). Maximum electron transport rate (*ETR*_max_), photosynthetic efficiency (α), and the saturating irradiance (*E*_k_) were significantly affected by CO_2_ and temperature. Optimal quantum yield for PSII fluorescence (*F*_v_/*F*_m_) was significantly affected by temperature and irradiance. All LL treatments showed *F*_v_/*F*_m_ values of ~0.75 (Table 1). HL caused a general decrease in *F*_v_/*F*_m_, which was stronger at 2 °C than at 7 °C, with values of ~0.39 and 0.67, respectively. *ETR*_max_ was significantly higher at 7 °C than at 2 °C. HL triggered a significant decrease of 30–40% at 2 °C, and a significant increase at 7 °C. Furthermore, elevated CO_2_ provoked a significant increase in *ETR*_max_ at 7 °C-HL. A similar response was obtained for α, with a significant increase at 7 °C-LL in comparison to 2 °C-LL, and a significant decrease at 2 °C-HL relative to 2 °C-LL. HL also caused a decrease in α at 7 °C at present CO_2_ conditions. *E*_k_ and the irradiance at which chronic photoinhibition begins (*E*_0pt_) were significantly altered by HL; this effect was stronger at 2 °C than at 7 °C, with values two-fold higher for *E*_k_ and three-fold higher for *E*_0pt_ at 2 °C-HL relative to LL conditions. Elevated CO_2_ provoked a decrease only in *E*_k_ at HL, regardless of the temperature.

**Table 1.** Photosynthetic parameters calculated from Chl a fluorescence measurements (mean±SD, n=6) of Desmarestia anceps after 12 days of culture at different CO_2_ levels (380 or 1000 ppm), temperatures (2 or 7 °C), and irradiance conditions (65 μmol photons m^−2^ s^−1^ or 145 μmol photons m^−2^ s^−1^)

		2 °C		7 °C	
		380 ppm CO _ 2 _	**1000 ppm CO**_**2**_	**380 ppm CO**_**2**_	**1000 ppm CO**_**2**_
*ETR*_max_ (μmol e^−^ m^−2^ s^−1^)	Low light	19.36 ± 1.79 ^a^	18.5 ± 1.91 ^a^	22.57 ± 0.87 ^b^	24.42 ± 0.86 ^bc^
	High light	11.93 ± 1.14 ^d^	13.94 ± 1 ^d^	25.29 ± 3.82 ^c^	27.57 ± 2.62 ^e^
α (e^−^ photons^−1^)	Low light	0.18 ± 0.02 ^ab^	0.17 ± 0.02 ^ab^	0.21 ± 0.02 ^bc^	0.22 ± 0.03 ^c^
	High light	0.05 ± 0.01 ^d^	0.07 ± 0.03 ^d^	0.15 ± 0.04 ^a^	0.22 ± 0.05 ^c^
*E*_k_ (μmol photons m^−2^ s^−1^)	Low light	107.1 ± 7.6 ^a^	105.9 ± 6.1 ^a^	109.4 ± 13.4 ^a^	110.9 ± 10.8 ^a^
	High light	251.4 ± 34.1 ^b^	201.7 ± 52.6 ^c^	172.2 ± 32.5 ^c^	129.9 ± 22.7 ^a^
*E*_0pt_ (μmol photons m^−2^ s^−1^)	Low light	214.1 ± 15.2 ^a^	221.7 ± 16.9 ^a^	253.2 ± 20 ^a^	277 ± 24.8 ^a^
	High light	601.5 ± 116 ^bd^	690.2 ± 190 ^d^	550.4 ± 62.4 ^bc^	492.4 ± 80.4 ^c^
*F*_v_/*F*_m_	Low light	0.75 ± 0.02 ^a^	0.74 ± 0.02 ^a^	0.75 ± 0.02 ^a^	0.76 ± 0.02 ^a^
	High light	0.38 ± 0.04 ^b^	0.41 ± 0.06 ^b^	0.65 ± 0.03 ^c^	0.69 ± 0.03 ^c^

Significant differences (*P*<0.05) revealed by Fisher’s LSD test following a three-way ANOVA (CO_2_, temperature, and light) are indicated by different letters.

Elemental composition was generally affected by temperature and by the interaction of irradiance and temperature, while it was not influenced by CO_2_, except for the FW:DW ratio. Total N content and C:N ratio were influenced by irradiance and by the interaction of CO_2_ and irradiance (Supplementary Table S2). Total C was significantly decreased at 2 °C-HL relative to 2 °C-LL at present CO_2_ conditions, whereas at elevated CO_2_ it was significantly higher at 7 °C-HL compared with 2 °C-HL (Table 2). Elevated temperature produced a significant decrease of 15–30% in the total N content. HL caused a significant increase in the total N content at 2 °C. Moreover, elevated CO_2_ significantly increased total N content at 2 °C-LL, while it produced a significant decrease at 7 °C-HL. The ^13^C isotopic discrimination in algal samples (δ^13^C_alga_) was significantly reduced at 7 °C relative to 2 °C. Elevated CO_2_ produced a significant decrease of δ^13^C_alga_ at 7 °C-HL, from -23.2 to -25.7‰. The FW:DW ratio showed a significant reduction of 10% at HL in comparison to LL conditions at 7 °C, while elevated CO_2_ provoked a significant decrease in FW:DW ratio at 2 °C-HL, from 5.39 to 4.72.

**Table 2.** Elemental composition of total C, total N, atomic C:N ratio, the corrected ^13^C isotopic discrimination in the algal samples (δ^13^C_alga_), and FW:DW ratio (mean±SD, n=6) of Desmarestia anceps after 12 days of culture at different CO_2_ levels (380 or 1000 ppm), temperatures (2 or 7 °C), and irradiance conditions (65 μmol photons m^−2^ s^−1^ or 145 μmol photons m^−2^ s^−1^)

		2 °C		7 °C	
		380 ppm CO _ 2 _	**1000 ppm CO**_**2**_	**380 ppm CO**_**2**_	**1000 ppm CO**_**2**_
Total C (% DW)	Low light	37.66 ± 2.1 ^ab^	37.08 ± 2.25 ^ab^	36.69 ± 1.02 ^a^	36.84 ± 1.27 ^ab^
	High light	34.65 ± 0.98 ^c^	36.74 ± 0.48 ^a^	37.81 ± 0.7 ^ab^	38.34 ± 0.76 ^b^
Total N (% DW)	Low light	3.4 ± 0.1 ^a^	3.52 ± 0.18 ^b^	2.83 ± 0.07 ^cd^	2.92 ± 0.08 ^cd^
	High light	4.1 ± 0.06 ^e^	4.04 ± 0.12 ^e^	2.94 ± 0.06 ^d^	2.82 ± 0.05 ^c^
C:N ratio	Low light	12.9 ± 0.52 ^a^	12.28 ± 0.52 ^b^	15.14 ± 0.7 ^c^	14.72 ± 0.31 ^c^
	High light	9.85 ± 2.26 ^d^	10.62 ± 0.41 ^e^	14.99 ± 0.17 ^c^	15.83 ± 0.14 ^f^
δ^13^C_alga_ (‰)	Low light	–19.79 ± 2.05 ^a^	–20.2 ± 2.66 ^a^	–23.24 ± 1.12 ^b^	–24.12 ± 2.03 ^bc^
	High light	–18.49 ± 1.13 ^a^	–18.25 ± 0.93 ^a^	–23.24 ± 1.93 ^b^	–25.65 ± 0.79 ^c^
FW:DW ratio	Low light	5.06 ± 0.56 ^ab^	4.98 ± 0.57 ^a^	4.88 ± 0.29 ^a^	4.74 ± 0.43 ^ac^
	High light	5.39 ± 0.14 ^b^	4.72 ± 0.13 ^a^	4.29 ± 0.22 ^c^	4.28 ± 0.19 ^c^

Significant differences (*P*<0.05) revealed by Fisher’s LSD test following a three-way ANOVA (CO_2_, temperature, and light) are indicated by different letters.

### Gene expression analysis

A total of 292553937 single-end reads were generated using the Illumina Hiseq platform. Reads per library ranged from 7.1 to 17.1 million, with an average of 12.2 million reads. Approximately 77.8% of the reads from all libraries mapped to the reference transcriptome, with an average of 9.5 million distinct alignments for each sample. Out of 53745 tested transcripts, 10663 (19%) showed significantly different regulation in at least one pairwise comparison. When comparing the total number of differentially expressed genes (DEGs) of the treatments against the control, the highest number of DEGs was observed in response to 2 °C-HLA (5440), followed by 2 °C-HLC (4555), whereas the rest of the treatments exhibited fewer than 1800 DEGs (Table 3). Pairwise comparisons across all treatments showed that increased CO_2_ conditions caused very small (72 DEGs at 2 °C) or no (0 DEGs at 7 °C) effects in HL-acclimated thalli, while elevated CO_2_ triggered a large number of DEGs (1295 at 2 °C and 954 at 7 °C) at LL conditions. HL induced a higher number of DEGs at 2 °C than at 7 °C, with 5440 DEGs at lower CO_2_ conditions and 2150 DEGs at elevated CO_2_ at 2 °C, whereas at 7 °C only 258 DEGs at lower CO_2_ conditions and 733 DEGs at elevated CO_2_ were obtained after comparison of both irradiance treatments (see Table 3).

**Table 3.** Number of significantly different up-regulated (upper right of the diagonal) and down-regulated (lower left of the diagonal, italics) transcripts in Desmarestia anceps after pairwise comparisons across all treatments

	**2-LLA**	**2-LLC**	**2-HLA**	**2-HLC**	**7-LLA**	**7-LLC**	**7-HLA**	**7-HLC**
**2-LLA**	—	682	2185	1651	745	181	671	953
**2-LLC**	*613*	—	1183	868	854	185	132	194
**2-HLA**	*3255*	*1598*	—	10	2739	2831	1301	1746
**2-HLC**	*2904*	*1282*	*62*	—	2375	2699	937	1409
**7-LLA**	*337*	*152*	*1620*	*1174*	—	131	23	67
**7-LLC**	*400*	*301*	*2262*	*1659*	*823*	—	267	366
**7-HLA**	*446*	*56*	*814*	*467*	*235*	*98*	—	0
**7-HLC**	*790*	*131*	*1272*	*844*	*679*	*367*	*0*	—

Up-regulated genes refer to the comparison of the treatments that appear in rows relative to the treatments that appear in columns, and down-regulated genes refer to the comparison of the treatments in columns relative to the treatments in rows. Genes were considered to be differentially expressed when the *P*-value was <0.01 and calculated absolute fold change between the control and the treatment was at least 2.

**Fig. 6.**
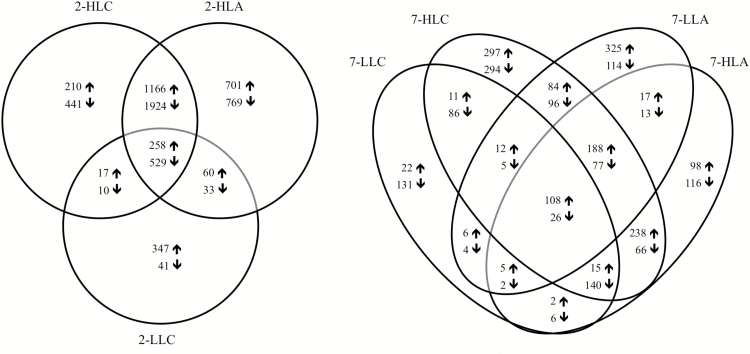
Venn diagram of differentially up-regulated (upward arrow) and down-regulated (downward arrow) transcripts in *Desmarestia anceps* after exposure to the different experimental conditions in comparison to the control (2-LLA). The number of regulated transcripts shared by the intersected treatments is shown for each intersection.

**Fig. 7.**
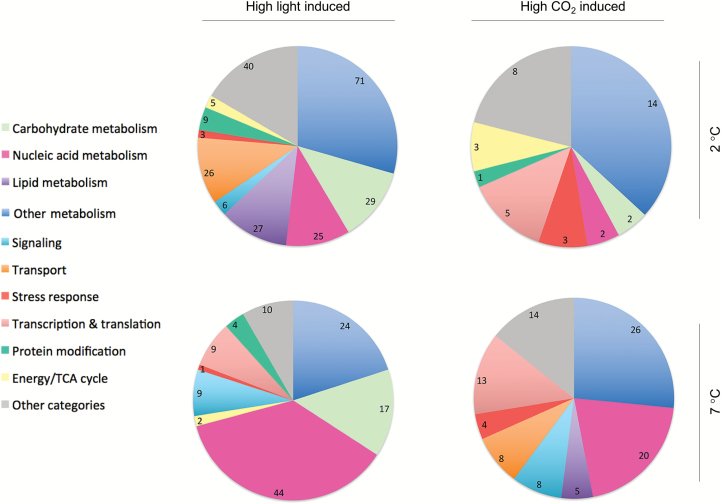
Relative distribution of putative functional categories derived from enriched GO terms of differentially expressed genes driven by high irradiance (intersection between HLA and HLC) or high CO_2_ (intersection between LLC and HLC) at the two tested temperatures (2 and 7 °C). All of them were compared against the control (2-LLA). Classification of enriched GO terms was made using cateGOrizer (EGAD2GO classification file). TCA, tricarboxylic acid.

A Venn diagram of all pairwise treatment versus control comparisons allowed the identification of an overlap of DEGs responsive to HL and/or high CO_2_ at the two different temperatures (Fig. 6). The number of DEGs involved in the HL-acclimation response decreased 10-fold with rising temperatures, from 3090 at 2 °C (intersection between 2-HLA and 2-HLC) to 304 at 7 °C (intersection between 7-HLA and 7-HLC). Elevated CO_2_ promoted a very low number of DEGs at both temperatures: 27 at 2 °C (intersection between 2-LLC and 2-HLC) and 97 at 7 °C (intersection between 7-LLC and 7-HLC). To cut down redundancies and assign biological processes to transcripts responding either to HL or elevated CO_2_, GO term enrichment analyses of the Venn diagram cross-sections described above were performed (Fig. 7). HL-acclimation caused a strong regulation of carbohydrate and nucleic acid metabolism-related transcripts at both temperatures. Furthermore, lipid metabolism and carrier proteins/membrane transport were highly regulated at 2-HL but not at 7-HL. Acclimation to elevated CO_2_ at both temperatures triggered a regulation of genes coding for transcription and translation and nucleic acid metabolism. At 2 °C, high CO_2_ caused a regulation of energy/tricarboxylic acid cycle-related transcripts, while at 7 °C, high CO_2_ provoked significant gene expression changes related to signalling and transport (Fig. 7).

Significant transcriptional changes of relevant transcripts encoding photosynthetic and carbon acquisition and assimilation components were analysed manually in comparison to the control (see Table 4). The majority of analysed transcripts coding for photochemical components, such as those from light-harvesting complexes (LHCs), oxygen-evolving complex, and chloroplastic electron transport chain, as well as proteins involved in chlorophyll biosynthesis, were 2- to 4.5-fold down-regulated only under 2 °C-HL conditions. The expression of transcripts encoding chloroplastic ATP synthase components was induced in all treatments at 7 °C except for 7-LLA. A transcript coding for carotene epsilon-monooxygenase was 2- to 3-fold up-regulated in all HL treatments. However, many genes encoding key proteins of the photochemical machinery were highly and constitutively expressed in all treatments (Supplementary Table S3), such as the PSII D1 protein, with the highest number of transcripts obtained for LHC components. A similar response was observed for the expression of genes related to the Calvin cycle, except for *RbcL*, with some of them showing a significant down-regulation (2.2–3.5-fold) only at 2 °C-HL and some others being constitutively expressed, but all of them showed a high level of expression (Supplementary Table S3). *RbcL* and *cfxQ* (encoding a putative Rubisco expression protein) were significantly up-regulated (2- to 4-fold) in all elevated CO_2_ treatments and under 7 °C-HL conditions (Table 4). However, other transcriptional regulators and post-transcriptional activators of Rubisco were constitutively expressed (Supplementary Table S3). With respect to CCM components, two transcripts coding for CAs (alpha and beta) and two genes encoding bicarbonate transporters were highly and constitutively expressed, except for one of the bicarbonate transporters (anion antiporter), which was down-regulated (2.3–2.4-fold) under 2 °C-HL conditions. High and constitutive expression of some genes coding for mitochondrial electron transport chain components, chloroplastic reactive oxygen species (ROS)-scavenging enzymes, and ferredoxin nitrite reductase was observed (Supplementary Table S3). A full list of differentially regulated transcripts against the control can be found in Supplementary Table S4.

**Table 4.** *Differentially regulated transcripts coding for photosynthetic-related components relative to the control (2-LLA*)

Gene ID	Putative gene product	Annotation e-value	Fold change						
			2-LLC	**2-HLA**	**2-HLC**	**7-LLA**	**7- LLC**	**7-HLA**	**7-HLC**
**Calvin cycle**									
Comp7666	Fructose-1,6-bisphosphatase	7e-143	—	–2.2	–2.4	—	—	—	—
Comp12243	Phosphoglycerate kinase	0	—	–3.5	–3.2	—	—	—	—
Comp15655	Phosphoribulokinase	6e-180	—	–3.1	–3.1	—	—	–2	—
Comp13735	Protein cfxQ homolog	1e-159	2.9	—	2.4	—	3.6	2.6	4.5
Comp13771	Ribulose bisphosphate carboxylase large chain	0	2.7	—	2.1	—	4	3.7	4.1
**Carbon-concentrating mechanism**									
Comp3434	Band 3 anion antiporter	3e-77	—	–2.3	–2.4	—	—	—	—
**Photochemical components**									
Comp7337	ATP synthase subunit alpha chloroplastic	0	—	—	—	—	3.7	2.5	4
Comp3494	ATP synthase subunit beta chloroplastic	0	—	—	—	—	3.8	3.1	3.9
Comp12802	Cytochrome b6-f complex iron-sulfur subunit	9e-79	—	–2.7	–2.6	—	—	—	—
Comp10525	Fucoxanthin-chlorophyll a-c binding protein D	1e-66	—	–3.4	–3	—	—	—	—
Comp10561	Fucoxanthin-chlorophyll a-c binding protein E	3e-36	—	–2.6	–2.3	—	—	—	—
Comp3360	Fucoxanthin-chlorophyll a-c binding protein F	2e-24	—	–3.1	–2.4	—	—	—	—
Comp4197	Ferredoxin	2e-18	—	–4.5	–4.1	—	—	—	—
Comp10540	Ferredoxin-NADP reductase	2e-140	—	–3.5	–3.4	—	—	—	—
Comp7594	Light-harvesting complex I LH38 proteins	2e-12	—	–3	–2.3	—	—	—	—
Comp16934	Photosystem I assembly protein Ycf4	2e-64	—	—	—	—	3.6	—	3.7
Comp11288	Photosystem II 12 kDa extrinsic protein (psbU)	2e-35	—	–2.4	–2	—	—	—	—
Comp14896	Photosystem II stability/assembly factor	5e-127	—	–3.7	–2.7	–2.2	—	–2.2	—
Comp19705	Protein PAM68	2e-14	—	–2.5	–2.6	—	—	—	—
Comp6464	Thylakoid luminal protein	7e-18	—	–3.1	–2.7	—	—	—	—
Comp11092	Oxygen-evolving enhancer protein 1 (psbO)	2e-61	—	–3.6	–3.3	—	—	—	—
Comp14031	Oxygen-evolving enhancer protein 3 (psbQ)	2.1e-08	—	–3.3	–3	—	—	—	—
**Others**									
Comp12786	Magnesium chelatase subunit ChlH	0	—	–2	–2.2	—	—	—	—
Comp14923	Carotene epsilon-monooxygenase	9e-26	—	3.1	3	—	—	2.1	2.1
Comp15426	Protochlorophyllide reductase	2e-106	—	–3.5	–3.4	—	—	—	—
Comp14384	Phosphoenolpyruvate carboxykinase (ATP)	0	—	–4.2	–3.5	—	—	—	—

All displayed genes were differentially expressed with *P*-values≤0.01 and were considered to be significant differently expressed with a fold change >2.

## Discussion

According to the results, sporophytes of *D. anceps* were highly benefited by the increase in temperature and CO_2_ when exposed to photoinhibitory irradiance for growth, although elevated temperature and CO_2_ did not significantly affect growth when exposed to non-photoinhibitory irradiance.

The growth rates reflect the strong cold and shade adaptation of this species and are in accordance with the results obtained by Wiencke and Fischer (1990). That study also showed that thalli cultured at 5 °C seemed to tolerate higher irradiances slightly better than those cultured at 0 °C. In the present study, the strong inhibition of growth at 2 °C-HL, which was not observed at 7 °C-HL, was accompanied by strong repression of transcripts encoding ribosomal components, such as 40S and 60S ribosomal proteins. This reduction in growth rate at 2 °C-HL can be explained by the fact that photosynthetic activity is particularly sensitive to low temperatures, as enzymatic secondary reactions are temperature-dependent (Q_10_ ~2–3), while primary light reactions are not (Raven and Geider, 1988). Thus, exposure to continuous high irradiance in combination with low temperature may result in an excess of electrons in the photosynthetic electron transport chain, leading to chronic photoinhibition (Maxwell *et al.*, 1994). These results agree with those of Heinrich *et al.* (2015), who found that higher temperatures seem to ameliorate the negative effects of UV radiation in sporophytes of *Saccharina latissima*. The increase in metabolic activity at higher temperatures was also reflected in a higher respiration rate at 7 °C, but only in HL-acclimated thalli. Furthermore, the increase in growth rate at elevated CO_2_ conditions in *D. anceps* is in accordance with the results obtained by Schoenrock *et al.* (2015) for natural populations at the beginning of their microcosm experiment, although they observed negative growth in all treatments during the last part of the 80-day incubation period, probably due to the long exposure to a constant photoperiod.

The increase in growth rates at elevated CO_2_ was paralleled by significantly higher gross photosynthetic rates. This response has been observed in other macroalgal species, and has been frequently related to DIC-limited thalli under current environmental conditions (Kübler *et al.*, 1999; Suárez-Álvarez *et al.*, 2012). Nevertheless, *D. anceps* seems to operate CCMs, as indicated by δ^13^C_alga_ values higher (less negative) than –30‰ (Raven *et al.*, 2002*b*), strong photosynthetic dependence on external CA activity, and high and constitutive expression of genes encoding CCM components. In addition, most polar macroalgae are known to possess the ability to actively use HCO_3_^−^ for photosynthesis (Wiencke and Fisher, 1990; Fisher and Wiencke, 1992; Beardall and Roberts, 1999). Assuming that the majority of polar macroalgae must be almost saturated at current CO_2_ conditions due to CCM operation along with the higher solubility of CO_2_ and a presumed increased S_C/O_ and CO_2_ affinity of Rubisco in cold waters, an absence of response of carbon fixation to increased CO_2_ might be expected, as shown by Young *et al.* (2015) for Antarctic phytoplankton and by Iñiguez *et al.* (2015) for Arctic seaweeds. Therefore, the increase in photosynthetic rates at elevated CO_2_ observed in the present study might correspond to an increase in Rubisco content, which is suggested by the induction of transcripts coding for *RbcL* at high CO_2_. The up-regulation of the *RbcL* gene under OA conditions was also observed in *P. tricornutum* (Li *et al.*, 2015), and Rubisco content increased at elevated CO_2_ in *T. pseudonana* and in *E. huxleyi* (McCarthy *et al.*, 2012). Conversely, other studies revealed a decrease in Rubisco content under OA conditions (García-Sánchez *et al.*, 1994; Andría *et al.*, 2001; Losh *et al.*, 2013) or no change in content (Israel and Hophy, 2002), suggesting the presence of species-specific differences.

The δ^13^C_alga_ values indicate that there was no significant down-regulation of CCM operation at elevated CO_2_, which is in accordance with the absence of regulation of genes coding for CCM components at high CO_2_, despite the observed decrease of ~20% in the photosynthetic dependence of external CA activity. Similarly, Trimborn *et al.* (2013) reported the operation of very efficient CCMs (possessing high inorganic C affinities) in four different Antarctic phytoplankton species that were not down-regulated after acclimation to elevated CO_2_ levels. It has been proposed that this lack of deactivation might be part of a mechanism that ensures high CO_2_ fuelling to Rubisco and prevents photoinhibition at low temperatures (Gordillo *et al.*, 2016). The observed strong DBS inhibition of photosynthesis provides evidence for the relevant role of external CA activity in inorganic carbon acquisition by *D. anceps*, in accordance with the elevated CA activities reported by Gordillo *et al.* (2006) for Arctic seaweeds, suggesting that this might be part of a general adaptation to cold waters. Comparable or just slightly higher photosynthetic inhibition promoted by EZ compared with DBS suggests a lower relevance of internal CA activity in inorganic carbon utilization. Similar results have been observed in previous studies with some members of the family Laminariaceae (Giordano and Maberly, 1989; Surif and Raven, 1989), whose CCMs are based on the simultaneous operation of proton pumps and periplasmic CA activity (Axelsson *et al.*, 2000; Klenell *et al.*, 2004). In *D. anceps*, inorganic carbon acquisition might also be based on external CA activity coupled with external proton extrusion, since an elevated number of genes coding for V-type proton ATPase components were found to be highly and constitutively expressed in all treatments. Direct bicarbonate uptake may represent another way of carbon incorporation in *D. anceps*, according to the expression of genes encoding bicarbonate transporters.

The more than 10-fold increase in DOC release rate of 2-HL-acclimated thalli, which corresponded to a release of 50% of the total assimilated carbon, is a strong evidence of physiological stress due to excessive light conditions (Sharp, 1977; Mague *et al.*, 1980). Accordingly, Chl *a* fluorescence measurements indicated a strong chronic photoinhibition, as reflected in a significant drop in the *F*_v_/*F*_m_ along with a low α and a significantly reduced *ETR*_max_ (Table 1). This response is indicative of photodamage (Hanelt *et al.*, 1997) and agrees with the down-regulation of photochemical and carbon utilization components at 2 °C-HL, which were mostly constitutively expressed in the rest of the treatments, leading to a downscaling of light harvesting as a response to high light stress. Moreover, the down-regulation of genes coding for proteins involved in chlorophyll biosynthesis (magnesium chelatase and protochlorophyllide reductase) is in accordance with the reduction in Chl *a* and *c* contents, suggesting accelerated pigment degradation under high light stress.

Acclimation to HL conditions was also reflected in a significant increase in *E*_k_ and *E*_0pt_ values. The increased light tolerance observed in HL-acclimated thalli was accompanied by significant induction of transcripts coding for carotene epsilon-monooxygenase, which is involved in xanthophyll biosynthesis, indicating a higher demand for photoprotection (Harker *et al.*, 1999). Furthermore, the constitutive high level of transcription of some ROS-scavenging enzymes in *D. anceps* might be related to its strong cold adaptation and might be involved in the ability of *D. anceps* to cope with short periods of exposure to high irradiance (Hanelt *et al.*, 1997), despite its strong shade adaptation.

In contrast to the effect on photosynthetic O_2_ evolution rates, increased CO_2_ conditions did not promote changes in the photochemical response of *D. anceps* except at 7 °C-HLC, at which there was a significant increase in *ETR*_max_ and α relative to 7 °C-HLA, which might be due to a photoprotective role of CO_2_ at elevated irradiance, as discussed above. A similar response was found for *D. tertiolecta*, which exhibited a higher physiological tolerance for excessive irradiance conditions at elevated CO_2_ compared with current CO_2_ levels (García-Gómez *et al.*, 2014). This photochemical response was not observed at 2 °C-HLC in comparison to 2 °C-HLA, probably due to a strong chronic photoinhibition in both treatments. Neither elevated CO_2_ nor HL conditions promoted an enhancement of the transcriptional expression of photochemical components such us LHCs, contrary to the transcriptomic results reported for *P. tricornutum* (Li *et al.*, 2015) and *E. huxleyi* (Rokitta *et al.*, 2012) after acclimation to OA, and to those obtained for the kelp *S. latissima* under high light stress (Heinrich *et al.*, 2015). Instead, *D. anceps* showed constitutive high expression of some LHC proteins, while others were significantly repressed only under 2 °C-HL conditions, suggesting that high and less regulated expression of proteins involved in photoprotection mechanisms might be part of the adaptation to cold environments.

A transcript encoding nitrite reductase was constitutively expressed at a high level in all treatments, which differs from the observed induction of nitrite reductase in *P. tricornutum* after acclimation to high CO_2_ conditions (Li *et al.*, 2015). This suggests that the assimilation of macronutrients other than CO_2_ might not be a genetically regulated process in *D. anceps*, and it can be related to the high nitrate concentrations that occur throughout the year in the Southern Ocean. However, the differences in total N between treatments must be due to post-translational regulation of nitrogen assimilatory enzymes, which seems to be uncoupled from CO_2_ assimilation.

The general strong regulation of carbohydrate metabolism under HL conditions suggests energy and redox metabolic reorganization due to excessive light exposure. Interestingly, light stress levels that did not lead to physiological alterations (7 °C-HL) caused a transcriptomic response, as shown by Heinrich *et al.* (2015) for *S. latissima*. Regulation of carbohydrate metabolism at elevated CO_2_, which was also reported by Rokitta *et al.* (2012) for *E. huxleyi*, was observed in the present study only at 2 °C and not at 7 °C. Conversely, high CO_2_ produced a regulation of signalling and transport at 7 °C but not at 2 °C, suggesting that the response of gene expression to increased CO_2_ is highly dependent on temperature, despite thalli acclimated to either temperature exhibiting the same physiological response, that is, an increase in photosynthesis and growth rate.

In conclusion, *D. anceps* would maintain its productivity in near-future scenarios of increased temperature and CO_2_ across a wider range of irradiance than under current conditions. The transcriptomic analysis revealed that gene expression of photosynthetic and carbon utilization components in *D. anceps* is less regulated than in other macrophytes in response to abiotic changes in temperature and CO_2_, which might be due to a strong adaptation to cold environments. This lack of genetic regulation might suggest a disadvantage with respect to cosmopolitan eurithermic species in near-future scenarios, since constitutively high gene expression requires extra energy that may be saved by species with more regulated gene expression in response to abiotic changes. Future experiments should focus on broad-scale community responses in order to confirm this response at the community level.

This is the first study to analyse the physiological and genetic responses of an ecologically relevant polar endemic macroalga to factors linked to global change. It provides a huge amount of transcriptomic information that could be used in future ecophysiological and metabolic studies.

## Supplementary data

Supplementary data are available at *JXB* online.

Table S1. Measurements of the seawater carbonate system over the experimental period.

Table S2. *P*-values of the three-way ANOVA for effects of temperature, CO_2_, irradiance, and their interaction on the physiological variables measured.

Table S3. Transcript per million counts of the control treatment corresponding to genes coding for photosynthetic-related components, most of them being constitutively expressed in all treatments.

Table S4. Full list of differentially regulated transcripts against the control treatment.

## Supplementary Material

supplementary_tables_S1_S3

supplementary_table_S4
