# Supplementary material for: Increased temperature and CO2 alleviate photoinhibition in Desmarestia anceps: from transcriptomics to carbon utilization
Source: J Exp Bot. 2017 Jun 1;68(14):3971–84. doi: 10.1093/jxb/erx164 (PMC5853390; doi:10.1093/jxb/erx164)
Supplement: supplementary_tables_S1_S3 [file erx164_suppl_supplementary_tables_s1_s3.pdf]

Supplementary data

Table S1. Seawater carbonate system of each treatment over the experimental period for *Desmarestia anceps*. Values are the mean ± standard deviation (SD). All replicates were analysed every 2-3 days (n = 6).

		**2ºC**		**7ºC**	
		**380 ppm CO_2**	**1000 ppm CO_2**	**380 ppm CO_2**	**1000 ppm CO_2**
pH_{NBS}	Low light	8.24 ± 0.01	7.8 ± 0.03	8.24 ± 0.03	7.84 ± 0.01
	High light	8.16 ± 0.01	7.77 ± 0.01	8.25 ± 0.02	7.86 ± 0.01
pCO_2 (µatm)	Low light	344 ± 6	1026 ± 56	348 ± 24	984 ± 22
	High light	410 ± 15	1076 ± 30	341 ± 21	927 ± 23
CO_2 (µmol kg SW^{-1})	Low light	19.2 ± 0.3	58 ± 3.1	16.6 ± 1.1	47 ± 1.1
	High light	23.2 ± 0.9	61.2 ± 1.7	16.2 ± 1	43.6 ± 1
HCO_3^- (µmol kg SW^{-1})	Low light	2153 ± 11	2355 ± 23	2101 ± 67	2297 ± 14
	High light	2142 ± 12	2293 ± 11	2058 ± 10	2291 ± 9
CO_3^{2-} (µmol kg SW^{-1})	Low light	122 ± 3	48 ± 3	141 ± 8	61 ± 2
	High light	100 ± 3	43 ± 1	141 ± 7	65 ± 2
DIC (µmol kg SW^{-1})	Low light	2295 ± 13	2462 ± 23	2231 ± 9	2404 ± 15
	High light	2266 ± 11	2398 ± 12	2216 ± 5	2400 ± 10
TA (µmol kg SW^{-1})	Low light	2451 ± 14	2473 ± 29	2414 ± 12	2443 ± 18
	High light	2387 ± 8	2400 ± 12	2400 ± 9	2450 ± 11

DIC, dissolved inorganic carbon; TA, total alkalinity.

Table S2. *P*-values of the three-way ANOVA for effects of temperature, CO_2, irradiance, and their interaction on the variables measured after 12 days of culture at the different CO_2 levels, temperatures and irradiance conditions for *Desmarestia anceps*. Statistically significant values are indicated by asterisks ($P < 0.05$).

	**CO_2**	**Irradiance**	**Temperature**	**CO_2 x Temperature**	**CO_2 x Irradiance**	**Irradiance x Temperature**	**CO_2 x Irradiance x Temperature**
Growth	<0.001*	<0.001*	<0.001*	0.192	0.485	<0.001*	0.051
Net photosynthesis	<0.001*	<0.001*	<0.001*	0.811	0.097	<0.001*	0.352
Respiration	0.002*	0.028*	0.268	0.533	0.451	<0.001*	0.068
Gross photosynthesis	<0.001*	<0.001*	<0.001*	0.471	0.180	<0.001*	0.073
DOC release	0.165	<0.001*	<0.001*	0.061	0.929	<0.001*	0.471
% DOC from assimilated C	0.009*	<0.001*	<0.001*	0.025*	0.001*	<0.001*	<0.001*
DBS inhibition of photosynthesis	<0.001*	<0.001*	0.016*	0.903	0.943	0.967	0.632
EZ inhibition of photosynthesis	<0.001*	<0.001*	0.190	0.006*	0.059	0.257	0.749
ETR_{max}	0.041*	0.019*	<0.001*	0.241	0.194	<0.001*	0.336
α	0.012*	<0.001*	<0.001*	0.125	0.028	<0.001*	0.595
E_k	0.005*	<0.001*	<0.001*	0.743	0.004	<0.001*	0.876
E_{0pt}	0.505	<0.001*	0.101	0.165	0.994	<0.001*	0.085
F_v/F_m	0.074	<0.001*	<0.001*	0.407	0.065	<0.001*	0.885
Total C	0.163	0.643	0.027*	0.588	0.055	<0.001*	0.148
Total N	0.803	<0.001*	<0.001*	0.460	0.001*	<0.001*	0.807
C:N ratio	0.243	<0.001*	<0.001*	0.572	<0.001*	<0.001*	0.816
$\delta^{13}C_{alga}$	0.086	0.388	<0.001*	0.121	0.656	0.020*	0.273
FW:DW ratio	0.045*	0.029*	<0.001*	0.162	0.306	0.013*	0.107
Chl a	0.885	<0.001*	<0.001*	0.578	0.511	<0.001*	0.002*
Chl c	0.085	<0.001*	<0.001*	0.781	0.604	<0.001*	0.008*
Total carotenoids	0.790	<0.001*	<0.001*	0.617	0.615	<0.001*	0.003*
Accessory pigments Chl a^{-1}	0.003*	0.174	0.227	0.067	0.105	0.080	0.731

Table S3. Transcript per million (TPM) counts of the control treatment (2-LLA) corresponding to genes encoding for photosynthetic and respiratory related components; most of them being constitutively expressed in all treatments.

**Transcript name**	**Putative gen product**	**Annotation e-Value**	**TPM**
Photochemical components			
Comp13674	D1 protein	0	304
Comp7563	Fucoxanthin-chlorophyll a-c binding protein C	5e-12	1366
Comp13412	Fucoxanthin-chlorophyll a-c binding protein B	7e-118	23913
Comp5287	Thylakoid formation protein	4e-29	138
Comp14026	ATP synthase gamma chain chloroplastic	2e-142	490
Comp13684	Photosystem I P700 chlorophyll a apoprotein	0	302
Comp7594	Light-harvesting complex I LH38 proteins	2e-12	1049
Comp10540	Ferredoxin-NADP reductase	2e-140	801
Comp12802	Cytochrome b6-f complex iron-sulfur subunit	9e-79	681
Comp11288	Photosystem II 12 kDa extrinsic protein (psbU)	2e-35	1885
Comp11092	Oxygen-evolving enhancer protein 1 (psbO)	2e-61	1503
Comp14031	Oxygen-evolving enhancer protein 3 (psbQ)	2.1e-08	1440
Calvin cycle			
Comp7556	Probable Rubisco transcriptional regulator	7e-109	196
Comp2931	Rubisco large subunit N-methyltransferase	1e-33	35
Comp17559	Protein cbbX homolog (Putative Rubisco activase-like)	3e-32	16
Comp13709	Glyceraldehyde-3-phosphate dehydrogenase	1e-164	1300
Comp13862	Sedoheptulose-1,7-bisphosphatase	2e-115	713
Comp12243	Phosphoglycerate kinase	0	722
Comp7666	Fructose-1,6-bisphosphatase	7e-143	377
Carbon concentrating mechanisms			
Comp3434	Bicarbonate transport protein (Band 3 anion antiporter)	3e-77	177
Comp3403	Sodium-driven chloride bicarbonate exchanger	4e-67	24
Comp4723	Beta carbonic anhydrase	1e-68	81
Comp13954	Alpha carbonic anhydrase	3e-22	148
chloroplastic ROS scavenging system			
Comp11295	Probable L-ascorbate peroxidase 6 chloroplastic	5e-101	147
Comp12715	Peroxiredoxin Q chloroplastic	5e-44	227
Comp11262	Peroxiredoxin-2E chloroplastic	4e-34	721
Respiration			
Comp13767	Cytochrome c oxidase subunit 6B1	3e-12	222
Comp15609	Cytochrome c oxidase biogenesis protein	2e-16	59
Comp3670	ATP synthase subunit O mitochondrial	8e-34	314

Comp14184	ATP synthase subunit delta mitochondrial	1e-29	224
Comp13736	ATP synthase subunit beta mitochondrial	0	534
Others			
Comp7055	Ferredoxin nitrite reductase	5e-149	77
Comp14384	Phosphoenolpyruvate carboxykinase (ATP)	0	690
Comp7327	Pyruvate, phosphate dikinase	0	113
Comp9842	NADP-dependent malic enzyme chloroplastic	0	50
